# Long-Term Effects of Angiotensin Receptor–Neprilysin Inhibitors on Myocardial Function in Chronic Heart Failure Patients with Reduced Ejection Fraction

**DOI:** 10.3390/diagnostics10080522

**Published:** 2020-07-28

**Authors:** Gregor Poglajen, Ajda Anžič-Drofenik, Gregor Zemljič, Sabina Frljak, Andraž Cerar, Renata Okrajšek, Miran Šebeštjen, Bojan Vrtovec

**Affiliations:** 1 Advanced Heart Failure and Transplantation Center, Department of Cardiology, University Medical Center Ljubljana, 1000 Ljubljana, Slovenia; ajda.drofenik@gmail.com (A.A.-D.); gregor.zemljic@kclj.si (G.Z.); sabina.frljak@kclj.si (S.F.); andraz.cerar@kclj.si (A.C.); renata.okrajsek@kclj.si (R.O.); miran.sebestjen@kclj.si (M.Š.); bojan.vrtovec@kclj.si (B.V.); 2 Department of Internal Medicine, Faculty of Medicine, University of Ljubljana, 1000 Ljubljana, Slovenia

**Keywords:** angiotensin receptor–neprilysin inhibitor, echocardiography, HFrEF

## Abstract

Background. We sought to evaluate the long-term effects of angiotensin receptor blocker–neprilysin inhibitor (ARNI) therapy on reverse remodeling of the failing myocardium in HFrEF patients. Methods. We performed a prospective non-randomized longitudinal study on 228 HFrEF patients treated with ARNI at our center. Prior to ARNI introduction all patients received stable doses of ACEI/ARB for at least six months. Clinical, biochemical and echocardiography data were obtained at ARNI introduction and 12-month follow-up. Results At follow-up, we found significant improvements in LVEF (29.7% ± 8% vs. 36.5% ± 9%; *p* < 0.001), LVOT-VTI (14.8 ± 4.2 cm vs. 17.2 ± 4.2 cm; *p* < 0.001), TAPSE (1.7 ± 0.5 cm vs. 2.1 ± 0.6 cm; *p* < 0.001) and LV-EDD (6.5 ± 0.8 cm vs. 6.3 ± 0.9 cm; *p* = 0.001). NT-proBNP serum levels also decreased significantly (1324 (605, 3281) pg/mL vs. 792 (329, 2022) pg/mL; *p* = 0.001). A total of 102 (45%) of patients responded favorably to ARNI (ΔLVEF < +5%; Group A) and 126 (55%) patients achieved ΔLVEF ≥ +5% (Group B). The two groups differed significantly in age, heart failure etiology, baseline LVEF and baseline NT-proBNP. On multivariable analysis, nonischemic heart failure, LVEF < 30% and NT-proBNP < 1500 pg/mL emerged as independent correlates of favorable response to ARNI therapy. Conclusion. ARNI therapy appears to improve echocardiographic parameters of left and right ventricular function in HFrEF patients above the effect of pre-existing optimal medical management. These effects may be particularly pronounced in patients with nonischemic heart failure, LVEF < 30% and lower degree of neurohumoral activation.

## 1. Introduction

With the publication of paradigm-HF trial (prospective comparison of ARNI with ACEI to determine impact on global mortality in heart failure) in 2014 [1] angiotensin receptor blocker–neprilysin inhibitors (ARNI) became a new promising class of drugs for the treatment of patients with heart failure with reduced ejection fraction (HFrEF). The study demonstrated that in HFrEF patient treatment with ARNI, compared to angiotensin converting enzyme inhibitor (ACEI) enalapril, resulted in significant benefits considering heart failure hospitalizations and cardiovascular (CV) and all-cause mortality [1]. Subsequent transition and pioneer-HF trials further demonstrated comparable safety and superior efficacy of ARNI over ACEI also in HFrEF patients with more advanced stages of the disease, largely establishing ARNI as an evolving first-line treatment approach in this patient population [2,3]. However, despite these encouraging findings, the underlying mechanisms still remain incompletely understood.

Studies of guidelines-based optimal heart failure medical therapy using angiotensin converting enzyme inhibitors (ACEI), angiotensin II receptor blockers (ARB), beta receptor blocking agents (β-blockers) and mineralocorticoid receptor antagonists (MRA) have demonstrated that improved clinical outcomes of HFrEF patients were associated with the reverse remodeling of the failing myocardium [4] which largely predicated on the inhibition of renin–angiotensin–aldosterone axis. Available data have suggested that in this patient cohort superior clinical response to ARNI over ACEI stems from a dual inhibition of renin–angiotensin–aldosterone axis and neprilysin, the latter resulting in an increased bioactivity of natriuretic and other vasoactive peptides [5]. However, until recently the association between ARNI-associated dual inhibition and myocardial reverse remodeling in HFrEF patients remained unexplored.

The recently published prove-HF trial confirmed the association of reverse remodeling and neurohumoral modulation in HFrEF patients treated with ARNI, demonstrating a significant correlation between changes in serum NT-proBNP and parameters of left ventricular volume and function in these patients [6]. While the results of several other small-scale reports are in line with prove-HF data [7,8,9], it is important to emphasize that the vast majority of presently available clinical data focus solely on the short-term effects of ARNI on left ventricular structure and function. In contrast, currently there are very few data on the long-term effects of ARNI therapy on myocardial reverse remodeling. What is more, no study to date addressed the effects of ARNI on the right ventricular function in HFrEF patients. This is relevant as in this patient cohort right ventricular dysfunction has been established as an important determinant of symptomatic limitations, cardiovascular outcomes and survival [10].

The aim of our study was therefore to evaluate the long-term effects of ARNI therapy on ventricular reverse remodeling in a HFrEF patient population.

## 2. Materials and Methods

### 2.1. Study Population

**Figure 1:**
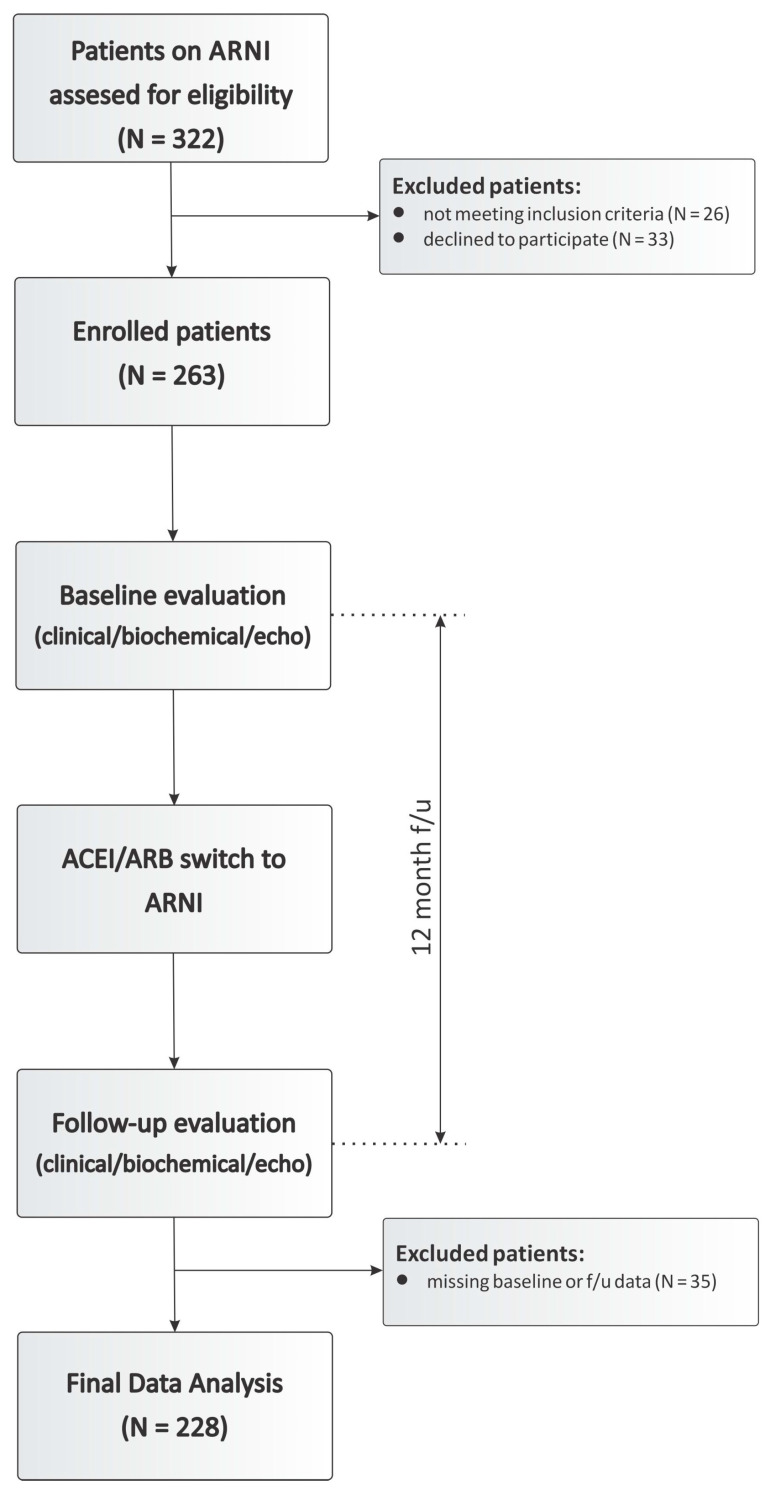
Study consort diagram.

We performed a single-center open-label prospective non-randomized longitudinal study to explore the effects of ARNI on the ventricular reverse remodeling in HFrEF patients (Figure 1). The study protocol was approved by the National medical ethics committee (decision document 0120–355/2017/6). All patients with HFrEF of ischemic and non-ischemic etiology that were treated at our center between years 2016 and 2019 were considered for the participation in the study. The inclusion criteria for the participation in the study were: patient age > 18 years, guideline-based heart failure medical management (OMT) for at least 6 months, established left ventricular systolic dysfunction (LVEF < 40%) and an established diagnosis of nonischemic dilated cardiomyopathy (DCMP) (as per European Society of Cardiology position statement [11]) or an established diagnosis of ischemic heart failure (ICM) without a possibility of percutaneous or surgical revascularization [12]. Exclusion criteria were as follows: patients with established heart failure with preserved ejection fraction (HFpEF), any hospitalization for severe worsening of heart failure (worsening heart failure requiring inotropic support) or acute myocardial infarction within 6 months before study enrollment, cardiac resynchronization therapy within 6 months before enrollment, patients with significant improvement of LVEF (ΔLVEF > +5% in 6 months prior to enrollment) on current optimal heart failure therapy and patients currently participating in other interventional studies. Of 322 assessed patients 26 did not meet the inclusion/exclusion criteria and 33 patients declined to participate in the study. Upon follow-up evaluation additional 35 patients were excluded due to the incomplete clinical, biochemical or echocardiographic data. Ultimately, 228 patients were included in the final data analysis. Informed consent was obtained from all patients before they were enrolled in the study.

### 2.2. Study Design

At enrollment, all patients were started on a sacubitril/valsartan dose per our institution’s protocol (Figure 1): if the patients were on an ACEI or ARB dose of at least 50% of target dose, they were started on an ARNI dose of 49/51 mg q12. Patients on an ACEI or ARB dose of less than 50% of target dose or with a history of hypotension (systolic blood pressure < 90 mmHg), liver or kidney insufficiency were initiated on an ARNI dose of 24/26 mg q12. ARNI uptitration was performed on weekly intervals if tolerated by the patient. During the study period patients maintained the doses of *β*-blockers and MRAs. Clinical, biochemical and echocardiographic data were collected at baseline and 12-month follow-up. Favorable response to ARNI therapy was defined as an increase in left ventricular ejection fraction (LVEF) ≥5% at 12-month follow-up [4].

### 2.3. Laboratory Tests

Laboratory tests (biochemistry, CBC, renal function tests and liver function test) were collected at baseline and at 12-month follow-up. All samples were collected in accordance with the institutional protocols and delivered to the institutional laboratory that was blinded to patient clinical data for the analysis.

### 2.4. NT-proBNP Measurement

Blood sample was collected at baseline and at 12-month follow-up. EDTA-coated, aprotinin-containing test tubes were used. Immediately after the blood sample was obtained, it was placed on ice (for up to 4 h) and centrifuged at 4500 rpm for 15 min at 0 °C. After centrifugation, the serum was extracted from the test tube and stored separately (at −80 °C). All NT-proBNP assays were done using a standard commercial kit (Roche Diagnostics, Rotkreuz, Switzerland) at a hospital’s central independent laboratory which was blinded to the patients’ clinical status and medication data.

### 2.5. Echocardiography

Transthoracic echocardiography was performed at baseline and 12 months according to American Society of Echocardiography (ASE) and European Association of Cardiovascular Imaging (EACVI) recommendations [13]. A cardiac ultrasound system (GE Vivid E9, Vingmed ultrasound, Horten, Norway) was used for imaging. All images were stored and analyzed at the end of the follow-up by an independent echocardiographer who was blinded to the patients’ therapeutic status and the timing of the recordings. Left ventricular function was assessed by left ventricular ejection fraction that was determined based on Simpson’s biplane rule. Right ventricular function was assessed by tricuspid annular plane systolic excursion (TAPSE).

### 2.6. Study End Points

The primary end point was a change in LVEF between baseline and 12-month follow-up. Secondary end points were the changes in TAPSE, left ventricular outflow tract velocity–time integral (LVOT VTI) and left ventricular end-diastolic diameter (LVEDD) between baseline and 12-month follow-up. In exploratory data analysis, we aimed to identify predictors of favorable response to ARNI therapy.

### 2.7. Statistical Methods and Analysis

Categorical variables are presented as count (percent) and were compared using a chi-squared test or nonparametric Fischer exact test. Continuous variables are reported either as mean (±SD) or median (IQR). Continuous variables were compared using Student’s *t*-test, ANOVA or Mann–Whitney nonparametric test. Pearson correlation model was used to assess the potential correlation between two continuous variables. Shapiro–Wilk test was used to test for the normality of data distribution. Statistical significance was assumed for *p*-values of <0.05. All statistical analyses were performed with SPSS version 20.0 (IBM, Chicago, IL, USA).

## 3. Results

### 3.1. Patient Characteristics

Baseline patient characteristics are outlined in Table 1. Majority of the patients included in our analysis were male with non-ischemic heart failure. Roughly half of the patients had a history of hypertension and hyperlipidemia and about a quarter of the patients had type 2 diabetes. The average LVEF and LVEDD were 30% and 6.5 cm, respectively. End-organ function was not significantly affected in any of the patients and no relevant biochemical abnormalities were registered. The baseline median (IQR) value of serum NT-proBNP was 1324 (605, 3281) pg/mL. At enrollment, all patients had been receiving maximal tolerated doses of optimal medical therapy. All patients received ACEI/ARB and β-blockers and 69% of patients also received MRAs. On average patient achieved 70% of recommended target dose of ACEI/ARB, 63% of recommended target dose of β-blockers and 95% of recommended target dose of MRA. While ACEI or ARB was switched for ARNI at enrollment, the doses of β-blockers and MRAs were not altered during the study period. The diuretic therapy was adjusted per discretion of the treating cardiologist. The average duration between baseline and follow-up visit in our patient cohort was 377 days.

### 3.2. Effects of ARNI on Ventricular Reverse Remodeling

**Figure 2:**
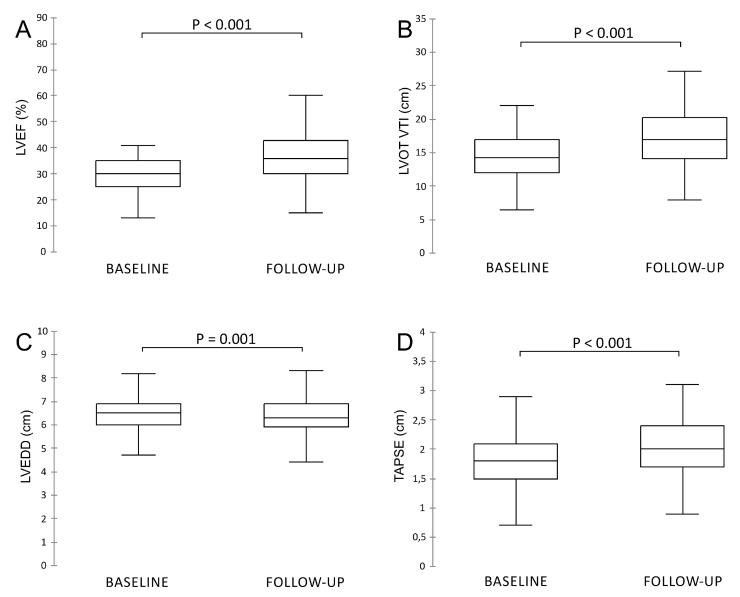
Compared to baseline, angiotensin receptor blocker–neprilysin inhibitor (ARNI) therapy showed significant improvement in (**A**) left ventricular systolic dysfunction (LVEF), (**B**) left ventricular outflow tract velocity–time integral (LVOT VTI) and (**C**) left ventricular end-diastolic diameter (LVEDD) and (**D**) tricuspid annular plane systolic excursion (TAPSE) at 12-month follow-up.

The effects of ARNI on the left and right ventricular reverse remodeling are outlined in Figure 2. Our results suggest that when in HFrEF patients ACEI or ARB therapy is switched to ARNI, this is associated with an additional improvement of left ventricular structure and function as we observed a significant increase in LVEF (29.7% ± 8% at baseline vs. 36.5% ± 9% at follow-up; *p* < 0.001) and LVOT VTI (14.8 ± 4.2 cm vs. 17.2 ± 4.2 cm; *p* < 0.001). At the same time, a decrease in LVEDD (6.5 ± 0.8 cm vs. 6.3 ± 0.9 cm; *p* = 0.001) was noted. Importantly, our data also showed that in this patient cohort ARNI therapy was associated with a significant improvement in right ventricular function as TAPSE changed from 1.7 ± 0.5 cm at baseline to 2.1 ± 0.6 cm (*p* < 0.001) at 12-month follow-up.

### 3.3. The Association of Neurohumoral Modulation and Reverse Remodeling

Our data also confirmed the beneficial effects of ARNI on neurohumoral modulation as NT-proBNP serum levels decreased significantly between baseline and 12-month follow-up (1324 (605, 3281) pg/mL vs. 792 (329, 2022) pg/mL; *p* = 0.001). However, we did not find a correlation between changes in NT-proBNP serum levels and LVEF (Pearson’s *r*^2^ = 0.01) or TAPSE (Pearson’s *r*^2^ = 0.003).

### 3.4. Response to ARNI and Reverse Remodeling

We further stratified patients according to the magnitude of the response to ARNI therapy (Table 2). We compared patients, in whom LVEF increased less than 5% in the 12-month follow-up period (Group A; *N* = 102, 45%) to patients, in whom an increase in LVEF of 5% or more was observed (Group B; *N* = 126, 55%). In Group A 50% of patients displayed a less than 5% increase of LVEF, in 6% of patients LVEF remained unchanged and in 44% of patients LVEF decreased. In the latter subgroup the mean decrease of LVEF was −4.6%. Comparing the two groups, we found significant differences in age (60 ± 10 years in Group A vs. 55 ± 11 years in Group B; *p* = 0.005), heart failure etiology (ischemic: 48% vs. 28%; *p* = 0.002), baseline LVEF (33% ± 7% vs. 27% ± 8%; *p* < 0.001) and baseline NT-proBNP 1612 (709, 3573) pg/mL vs. 1112 (513, 3027) pg/mL; *p* = 0.03). Our data also showed that more patients in Group A received low dose of ARNI (22% vs. 12%; *p* = 0.03), while there were no differences regarding the intermediate (38% vs. 39%; *p* = 0.94) and high (40% vs. 49%; *p* = 0.47) ARNI doses between the two groups. On multivariable analysis, nonischemic heart failure, baseline LVEF < 30% and baseline NT-proBNP serum levels less than 1500 pg/mL emerged as independent predictors of favorable response to ARNI therapy (Table 3).

**Figure 3:**
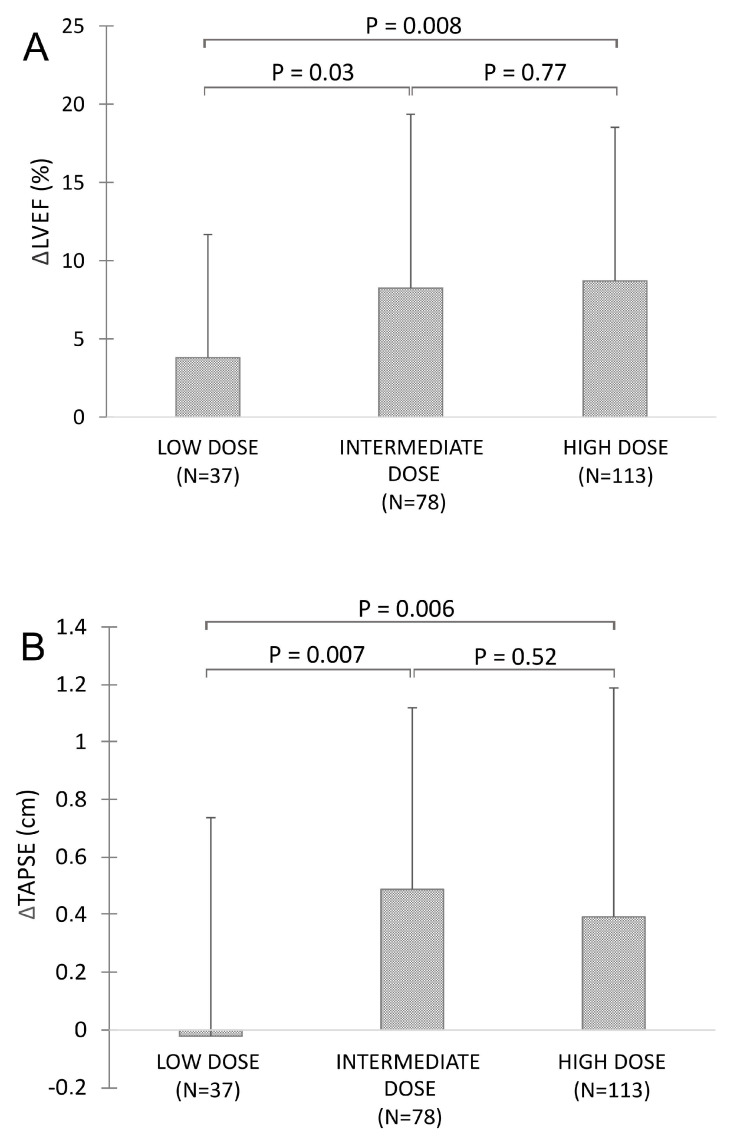
Effect of ARNI dose on (**A**) LVEF and on (**B**) TAPSE.

Evaluation of the response to ARNI therapy according to the ARNI dose (Figure 3) showed that the low dose of ARNI was associated with lower increases in LVEF (+3.8% ± 7.2%) and TAPSE (−0.1 ± 0.7 cm) than intermediate (LVEF: +8.2% ± 11.1%; TAPSE +0.5 ± 0.6 cm) or high doses of ARNI (LVEF: +8.7% ± 9.8%; TAPSE +0.4 ± 0.7 cm).

## 4. Discussion

The current analysis suggests that in HFrEF patients previously treated with maximal tolerated doses of guideline-based medical therapy a switch to ARNI-based medical regimen may further promote reverse remodeling of the failing myocardium and that this response is likely dose-dependent. Our data additionally suggest that these beneficial effects are particularly pronounced in patients with non-ischemic heart failure, with lower baseline LVEF and with less prominent baseline neurohumoral activation.

In the past 20 years, four major drug classes (ACEI, ARB, β-blockers and MRAs) were introduced for the treatment of HFrEF. Inhibiting the renin–angiotensin–aldosterone axis they have significantly improved the morbidity and mortality of these patients [14,15,16,17]. Importantly, the beneficial effects of these medical therapies were repeatedly associated with their potential to promote myocardial reverse remodeling manifested mainly by the reduction of left ventricular size and improvement of its function [18]. While paradigm-HF and pioneer-HF data clearly demonstrated superior clinical efficacy of ARNI therapy compared to ACEI in terms of reduced heart failure-associated hospital admissions, cardiovascular and all-cause mortality, until recently the association between these clinical effects and reverse remodeling of the failing myocardium remained unexplored [1,2]. Basing largely on the small patient cohorts and short-term follow-up recently published data support the association between ARNI and left ventricular reverse remodeling as these studies consistently showed an improvement in left ventricular ejection fraction (ΔLVEF +4–5%) and a decrease in left ventricular size (ΔLVEDD—6% or ΔLVEDV—8–10%) after three months of ARNI therapy [7,8,9]. A large prove-HF trial corroborated these initial findings and further established long-term beneficial effects of ARNI therapy on left ventricular reverse remodeling as significant improvements in left ventricular ejection fraction (ΔLVEF +9.4%) and size (ΔLVEDV–14%) were established 12 months after the initiation of ARNI therapy [6]. Apart from prove-HF trial our study is the only prospective study to date evaluating long-term effects of ARNI therapy on myocardial reverse remodeling in HFrEF patient population. Our results are in line with the prove-HF data as we have also established comparable long-term improvements in left ventricular size (ΔLVEDD–4% and function (ΔLVEF +6.8%) in HFrEF patients receiving ARNI therapy. Taken together, these data show that in the HFrEF patient population ARNI therapy may lead to early and, importantly, lasting improvement in myocardial reverse remodeling.

While emerging data suggest a significant benefit of ARNI therapy on left ventricular reverse remodeling there are currently almost no data available on the effects of ARNI on right ventricular function in HFrEF patient population. Bayard et al. failed to establish any benefit of ARNI therapy on right ventricular function in 52 patients with HFrEF as no changes in TAPSE were observed throughout the study period (3 months) [9]. Contrary to these conclusions our data suggest that ARNI therapy may be associated with an improvement of right ventricular function as TAPSE improved significantly after 12 months of ARNI therapy in our patient cohort. This differences in results could partly be attributed to the differences in sample size as the study of Bayard et al. was likely underpowered to adequately evaluate the effects of ARNI therapy on TAPSE. Additionally, prove-HF trial, while not specifically evaluating right ventricular function, indirectly supports our results as it demonstrated positive effects of ARNI therapy on left ventricular diastolic function showing a significant decrease in left atrial volume index and left ventricular end-diastolic filling pressure (E/e′) at 12-month follow-up [6]. These observations were further supported by Mullens et al. demonstrating that ARNI therapy may result in a prolonged left ventricular diastolic filling time and in a significant reduction in number of patients demonstrating restrictive mitral filling pattern [8]. Collectively, these results may suggest that ARNI therapy could reduce right ventricular afterload indirectly through the improvement of left ventricular diastolic function. This in combination with the effects of ARNI-induced dual neurohumoral blockade on intravascular volume reduction (right ventricular preload reduction) and anti-inflammatory and anti-fibrotic effects that ARNI therapy exerts on the failing myocardium may explain an improved right ventricular systolic function, established in our trial [19]. This could also translate into clinical relevance as right ventricular dysfunction has been established as an important determinant of symptomatic limitations, cardiovascular outcomes and survival in HFrEF and HFpEF patients alike [10].

In accordance with previously published literature [1,2,19] our data also confirmed the beneficial effects of ARNI on neurohumoral modulation as NT-proBNP serum levels decreased significantly between baseline and 12-month follow-up, supporting the dual neurohumoral blockade as a primary pathophysiological mechanism of ARNI therapy. However, we have failed to establish a correlation between changes in NT-proBNP serum levels and changes in left ventricular ejection fraction or TAPSE, respectively. This finding contrasts the conclusion of prove-HF trial, where a weak, but significant correlation was established between reduction in NT-proBNP and improvements in markers of left ventricular structure and function [6]. Several explanations may be offered to justify this discrepancy: (1) whereas the correlation between changes NT-proBNP concentrations and markers of left structure and function were the primary end-point of prove-HF, this was a subject of exploratory analysis in our trial; (2) the population in the prove-HF trial was more than three times the size of the population included in our analysis; and (3) our patient cohort was roughly 10 years younger, had higher prevalence of male patients, much higher baseline NT-proBNP serum levels and significantly higher percentage of patients treated with MRAs. Any of these differences could confound a potential association between changes in serum NT-proBNP and changes in structure and function of left or right ventricle in our patient cohort.

Considering the response to ARNI therapy our data suggest that patients with non-ischemic heart failure, patients with left ventricular ejection fraction less than 30% and with less pronounced neurohumoral activation may display particularly good response to ARNI therapy. In terms of left ventricular ejection fraction and neurohumoral activation these results are supported by paradigm-HF data that showed better clinical response to ARNI therapy in patients with lower left ventricular ejection fraction and with NT-proBNP levels below the median value [1]. While this may seem counterintuitive, these findings may be explained by an elegant study of Gremmler et al. who showed that in stable/compensated HFrEF patients NT-proBNP serum levels appear to be highest with LVEF between 30–40% (around 2000 pg/mL). In patients with LVEF between 15% to 30%, NT-proBNP serum levels decreased to around 1500 pg/mL [20]. While pathophysiological background of this remains inadequately explained it is speculated that HFrEF patients with LVEF between 30 and 40% may develop higher wall tension in the failing myocardium, so the stimulus for natriuretic peptide excretion could be more pronounced in this patient cohort. Similarly, in the field of cardiac resynchronization therapy (CRT) REVERSE study investigators showed that patients with left ventricular ejection fraction < 30% displayed significant improvements in clinical response to CRT as well as in echocardiographic parameters of left ventricular reverse remodeling that may be even more pronounced than in patients with left ventricular ejection fraction > 30% [21]. Our data also suggest better response to ARNI therapy in patients with non-ischemic heart failure as left ventricular ejection fraction improved for +9.4% in patients with non-ischemic heart failure and for +4.5% in patients with ischemic heart failure. This may seem intuitive as impaired coronary perfusion may significantly diminish the capacity of the failing myocardium to recover its structure and/or function. Nevertheless, paradigm-HF sub-analysis demonstrated similar clinical benefits in patients with ischemic and non-ischemic heart failure receiving ARNI therapy [22]. It can be argued that a relative increase in left ventricular ejection fraction of +4.9% in favor of patient with non-ischemic heart failure seen in our data are likely not sufficient to translate to meaningful differences in clinical outcomes between patients with ischemic and non-ischemic heart failure.

Finally, in our patient cohort a dose-dependent effect of ARNI therapy was noted as an increase in left ventricular ejection fraction and TAPSE was significantly lower in patients receiving low dose of ARNI than in patients receiving intermediate or high doses of ARNI. We did not observe any differences in response to ARNI between the groups of patients receiving intermediate or high ARNI doses. These observations are in line with the data from Mullens et al. who also demonstrated dose-dependent effect of ARNI therapy [8]. Importantly, a similar dose-dependent effect has been noted for most the heart failure therapies to date [18,23], again stressing the importance of heart failure therapy titration in HFrEF patient population. Interestingly, in prove-HF trial this dose-dependent effect was blunted, likely due to the fact that 67% of the prove-HF patient population reached the high ARNI dose and most the remaining 33% of patients reached the intermediate dose of ARNI [6].

Our study has several limitations that must be acknowledged. First is its single-group, open-label design. However, this study design may be justified due to a widespread bioavailability of ARNI, its class I clinical practice guideline recommendation and proven superiority over ACEI/ARB in most the HFrEF patients. Second, compared to some recently published heart failure trials, there may be an underrepresentation of the female gender in our study. Third, the size of our study sample—despite being second only to prove-HF considering the studies analyzing echocardiographic response to ARNI therapy—is still relatively small. Therefore, our study was likely not sufficiently powered for some performed analyses, such as a correlation between changes in serum NT-proBNP and left ventricular ejection fraction or TAPSE. Fourth, while many more informative and intricate echocardiographic parameters could be assessed, we chose only the most robust and easily accessible ones for our analysis in order to make our conclusions applicable not only to the tertiary centers with access to the advanced imaging technology, but also to the general cardiology practices. We acknowledge that adding other echocardiographic parameters to the analysis may add novel insights into the reverse-remodeling in the HFrEF patient population and fully support the verification of our preliminary data in such a trial.

## 5. Conclusions

ARNI therapy appears to promote long-term reverse remodeling of both left and right ventricle in HFrEF patient populations, above and beyond the effect of pre-existing optimal medical management. These effects may be particularly pronounced in patients with nonischemic heart failure, LVEF < 30% and lower degree of neurohumoral activation.

## Figures and Tables

**Table 1:** Baseline patient characteristics.

Variable	Baseline (*N* = 228)	Follow-Up (*N* = 228)	*p*
Age, y	57 ± 11		/
Male gender (%)	189 (83)		/
Ischemic heart failure (%)	82 (36)		/
Sodium, mmol/L	140 ± 2	141 ± 3	0.78
Potassium, mmol/L	4.7 ± 0.5	4.8 ± 0.4	0.45
Creatinine, µmol/L	95 ± 34	97 ± 38	0.48
Bilirubin, µmol/L	17 ± 13	16 ± 10	0.28
NT-proBNP, pg/mL (IQR)	1324 (605, 3281)	792 (329, 2022)	0.001
Comorbidities			
Hypertension (%)	123 (54)		/
Diabetes (%)	52 (23)		/
Hyperlipidemia (%)	129 (57)		/
Chronic kidney disease (%)	56 (24)		/
Atrial fibrillation (%)	67 (29)		/
Baseline medical therapy			
ACEI/ARB (%)	228 (100)	0	/
% of target dose	70	0	/
ARNI (%)	0	228 (100)	/
% of target dose	0	69	/
Beta blockers (%)	228 (100)	228 (100)	/
% of target dose	63	63	/
MRA (%)	157 (69)	157 (69)	/
% of target dose	100	100	/
Digoxin (%)	23 (10)	27 (12)	0.58
ICD/CRT (%)	64 (28)	64 (28)	/

Legend: ACEI—angiotensin converting enzyme inhibitor; ARB—angiotensin receptor blocker; ARNI—angiotensin receptor blocker/neprilysin inhibitor; ICD—implantable cardioverter defibrillator; CRT—cardiac resynchronization therapy.

**Table 2:** Baseline characteristics of patients according to the response to ARNI therapy.

Variable	Group A (*N* = 102)	Group B (*N* = 126)	*p*
Age, y	60 ± 10	55 ± 11	0.005
Male gender (%)	84 (82)	106 (84)	0.97
Ischemic heart failure (%)	49 (48)	35 (28)	0.002
Creatinine, µmol/L	100 ± 39	95 ± 37	0.39
Bilirubin, µmol/L	16 ± 8	15 ± 12	0.74
NT-proBNP, pg/mL (IQR)	1612 (709, 3573)	1112 (513, 3027)	0.03
LVEF (%)	33 ± 7	27 ± 8	<0.001
LVEDD, cm	6.5 ± 0.9	6.5 ± 0.7	0.75
TAPSE, cm	1.7 ± 0.5	1.7 ± 0.4	0.78
Comorbidities			
Hypertension (%)	59 (58)	63 (50)	0.20
Diabetes (%)	27 (26)	26 (21)	0.41
Hyperlipidemia (%)	62 (61)	67 (53)	0.19
Chronic kidney disease (%)	24 (23)	32 (25)	0.89
Atrial fibrillation (%)	32 (31)	35 (28)	0.76
Baseline medical therapy			
ACEI/ARB (%)	102 (100)	126 (100)	/
Beta blockers (%)	102 (100)	126 (100)	/
MRA (%)	85 (83)	93 (74)	0.86
Digoxin (%)	12 (12)	11 (9)	0.53
ICD/CRT (%)	34 (33)	29 (23)	0.10
ARNI dose			
Low dose (%)	22 (22)	15 (12)	0.03
Intermediate dose (%)	39 (38)	49 (39)	0.94
High dose (%)	41 (40)	62 (49)	0.47

**Table 3:** Multivariable analysis of predictors of response to ARNI therapy.

Variable	B	*p*	95% Confidence Interval	
			Lower Bound	Upper Bound
Age > 60 years	−0.129	0.713	0.443	1.745
Ischemic heart failure	−0.699	0.044	0.252	0.981
LVEF > 30%	−1.711	0.001	0.087	0.374
NT-proBNP > 1500 pg/mL	−0.813	0.035	0.208	0.945
Low-dose ARNI therapy	−0.588	0.232	0.212	1.456
